# Visualization and tracking of tumour extracellular vesicle delivery and RNA translation using multiplexed reporters

**DOI:** 10.1038/ncomms8029

**Published:** 2015-05-13

**Authors:** Charles P. Lai, Edward Y. Kim, Christian E. Badr, Ralph Weissleder, Thorsten R. Mempel, Bakhos A. Tannous, Xandra O. Breakefield

**Affiliations:** 1 Department of Neurology and Radiology, Massachusetts General Hospital, and Program in Neuroscience, Harvard Medical School, 149 13th Street, Charlestown, Massachusetts 02129, USA; 2 Center for Immunology and Inflammatory Diseases and Department of Medicine, Massachusetts General Hospital, and Program in Immunology, Harvard Medical School, Charlestown, Massachusetts 02129, USA; 3 Center for Systems Biology, Department of Radiology, Massachusetts General Hospital, and Department of Systems Biology, Harvard Medical School, Boston, Massachusetts 02114, USA

## Abstract

Accurate spatiotemporal assessment of extracellular vesicle (EV) delivery and cargo RNA translation requires specific and robust live-cell imaging technologies. Here we engineer optical reporters to label multiple EV populations for visualization and tracking of tumour EV release, uptake and exchange between cell populations both in culture and *in vivo*. Enhanced green fluorescence protein (EGFP) and tandem dimer Tomato (tdTomato) were fused at NH_2_-termini with a palmitoylation signal (PalmGFP, PalmtdTomato) for EV membrane labelling. To monitor EV-RNA cargo, transcripts encoding PalmtdTomato were tagged with MS2 RNA binding sequences and detected by co-expression of bacteriophage MS2 coat protein fused with EGFP. By multiplexing fluorescent and bioluminescent EV membrane reporters, we reveal the rapid dynamics of both EV uptake and translation of EV-delivered cargo mRNAs in cancer cells that occurred within 1-hour post-horizontal transfer between cells. These studies confirm that EV-mediated communication is dynamic and multidirectional between cells with delivery of functional mRNA.

## Abstract (web summary)

Extracellular vesicles (EVs) act as a conduit for intercellular communication through the exchange of cellular materials without direct cell-to-cell contacts. Here the authors develop a multiplexed reporter system that allows monitoring of EV exchange, cargo delivery and protein translation between different cell populations.

## Graphical abstract

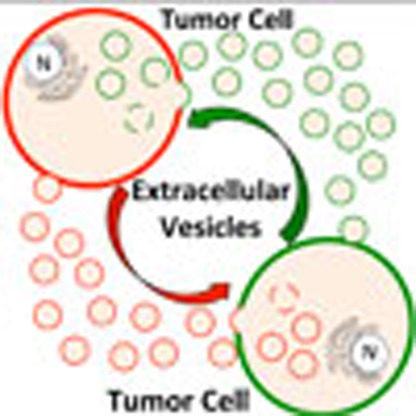

Cells release exosomes, microvesicles and apoptotic blebs as a conduit for intercellular communication without direct cell-to-cell contacts^1^,^2^,^3^,^4^ {Colombo:2013bt, Camussi:2010uk, Vader:2014fq, ELAndaloussi:2013ca}-CITATION_IS_EMPTY. These vesicles, collectively termed extracellular vesicles (EVs), are capable of horizontal transfer of lipids, (glyco)proteins and nucleic acids from EV-donor cells to neighbouring and/or distal recipient cells^5^,^6^. Previous reports support the functionality of EV-delivered mRNAs^7^,^8^, but direct visualization of RNA cargo and mRNA fate has been lacking, essential in understanding the kinetics and spatiotemporal arrangements. While different methods including electron microscopy and atomic force microscopy can be used to study the physical properties of EVs, live-cell imaging at high spatial resolution generally requires fluorescent microscopy techniques using EV-associated proteins fused to fluorescent proteins, and/or fluorescent chemical labelling^6^,^9^,^10^,^11^,^12^.

Current EV labelling methods have some limitations. For fluorescent proteins conjugated to proteins enriched in EVs, labelling may be restricted to subpopulations of EVs, limiting their wider use to observe multiple EV types. In contrast, fluorescent dyes for EV lipid labelling including PKH may not reflect true half-life of EVs and can be retained in association with other lipid entities for long periods, thus misguiding spatiotemporal assessment of EV dynamics, especially over extended periods^13^. In addition, studying the fate of mRNAs transcribed and packaged into EVs released by cells has been challenging due to their low quantity^14^, thus demanding a highly sensitive imaging technique. Furthermore, limitations on EV reporter choice render current studies on EV-mediated communication mainly to unidirectional EV transfer from one cell type to another^2^, whereas EV exchange under physiological and/or pathological condition is expected to be multidirectional between cell populations.

*S*-Palmitoylation through a thioester linkage between the sulfhydryl group on cysteines and the fatty acid palmitic acid enables association of proteins with cellular membranes^15^. By fusing fluorescent proteins with a consensus palmitoylation sequence, these reporters can be transferred to cellular membrane, enabling whole-cell labelling^16^,^17^. As EVs are derived from the plasma membrane either via inward invagination of endosomes to form multivesicular bodies releasing exosomes^18^ or outward budding and shedding for microvesicles and apoptotic blebs released from the cell surface^19^,^20^,^21^, we hypothesized that tagging the plasma membrane with fluorescent proteins would enable labelling of multiple EV types.

Bioluminescence reporters have also been widely adapted to study biological processes including gene expression based on their sensitivity and low background signal without the need of an excitation source to emit light^22^. Specifically, *Gaussia* luciferase (Gluc), which is over 1,000-fold brighter than commonly used luciferases^23^, catalyses flash bioluminescence with rapid signal decay, making it an ideal reporter to study temporal properties of minute events, such as translation of EV-delivered mRNAs.

In this study, we develop a fluorescent EV labelling strategy to achieve live-cell imaging of EV release, uptake and exchange between different cell populations, as well as microscopic quantification and flow cytometry analysis. By multiplexing with the bacteriophage MS2 coat protein (MS2CP) mRNA fluorescent reporter system^24^ and a bioluminescent EV reporter^13^, we visualize EV-mRNA cargo and further reveal the temporal dynamics of EV uptake and translation of EV-delivered mRNAs.

## Results

### PalmGFP and PalmtdTomato label EVs

**Figure 1:**
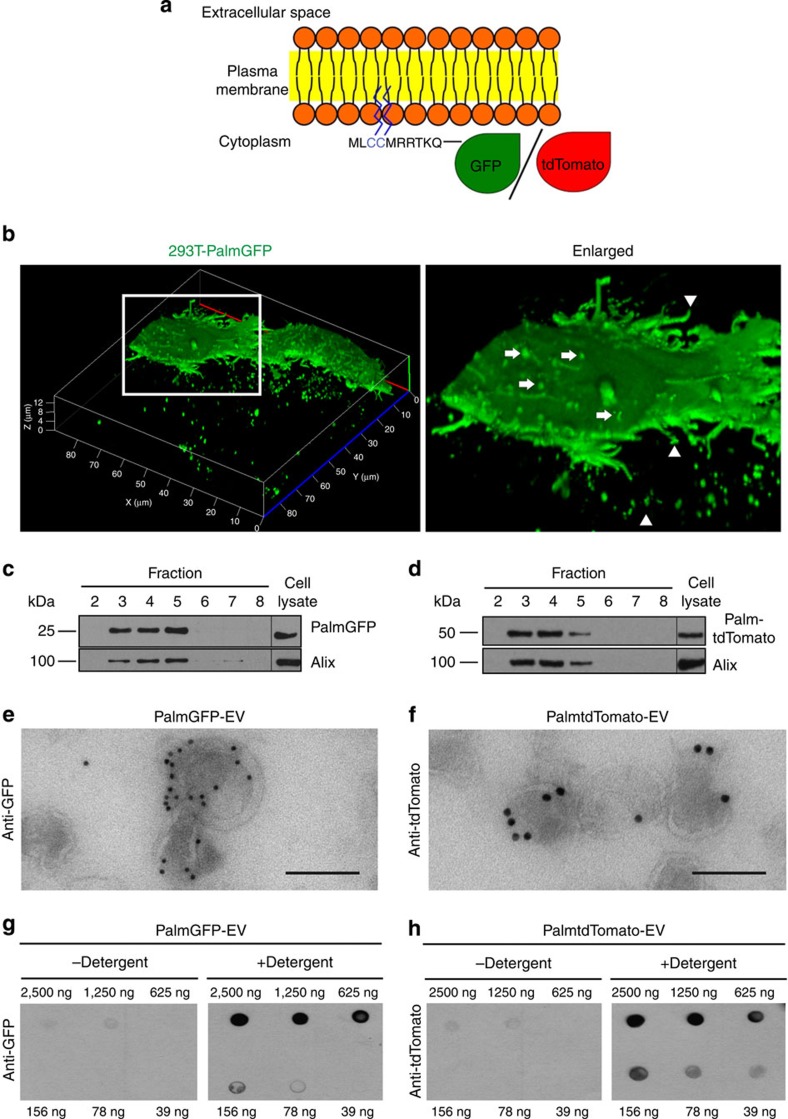
Palmitoylated-GFP and tdTomato are trafficked to the plasma membrane and EVs. (**a**) Schematic diagram of EV membrane labelling with palmitoylated GFP (PalmGFP) or tdTomato (PalmtdTomato). (**b**) Live-cell confocal microscopy of stable PalmGFP-expressing 293T cells releasing EVs. Left panel: 3D reconstruction of confocal Z-stack images demonstrating EV release from stable 293T-PalmGFP cells into surrounding regions. Right panel: enlarged image of boxed region, showing bud-like structure from the surface (arrows) as well as processes extending from cells (arrow heads). (**c**,**d**) Western blot analysis of protein extracted from fractions following sucrose-gradient centrifugation of isolated EVs. PalmGFP (**c**) and PalmtdTomato (**d**) were detected in fractions exhibiting the EV marker, Alix (95 kDa). (**e**,**f**) Transmission electron micrograph (TEM) showing PalmGFP and PalmtdTomato labelling of EVs on the membrane following immunolabelling with anti-GFP (**e**) or anti-tdTomato (**f**) and secondary gold-conjugated secondary antibodies. Scale bar, 100 nm. (**g**) Demonstration that PalmGFP and PalmtdTomato labels the inner membrane of EVs. PalmGFP- or PalmtdTomato EVs were dot blotted on nitrocellulose membrane in a dose range followed by immunoprobing with anti-GFP or anti-tdTomato, respectively, and horseradish peroxidase (HRP)-conjugated secondary antibodies in the presence or absence of detergent [0.1% (v/v) Tween20] for chemiluminescence detection.

To generate fluorescent EV reporters, a palmitoylation signal was genetically fused in-frame to the N terminus of EGFP (PalmGFP)^16^,^17^ or tdTomato^25^ (PalmtdTomato) (Fig. 1a). 293T cells were then stably transduced with a lentivirus vector encoding either PalmGFP (293T-PalmGFP) or PalmtdTomato (293T-PalmtdTomato) to examine EV reporter expression. Live-cell confocal microscopy of 293T-PalmGFP cells showed that PalmGFP uniformly labels the plasma membrane and reveals budlike structure on their surface and processes (Fig. 1b). PalmGFP^+^ EVs of varying sizes were also detected around 293T-PalmGFP cells, suggesting the reporter labels multiple EV subtypes (Fig. 1b). To verify specificity of the EV reporters, EVs isolated from 293T-PalmGFP or 293T-PalmtdTomato cells were isolated by sucrose density gradient centrifugation, and PalmGFP and PalmtdTomato labels were found specifically in fractions 3–5, which are also the fractions exhibiting the exosomal/microvesicle marker Alix^26^,^27^ (Fig. 1c,d; Supplementary Fig. 1). To further examine whether PalmGFP or PalmtdTomato labels EVs in association with membranes, transmission electron microscopy was applied in combination with immunogold labelling of PalmGFP or PalmtdTomato. These experiments showed that EVs were indeed labelled on their membranes, and not within the EVs (Fig. 1e,f, Supplementary Fig. 2). To characterize the orientation of PalmGFP and PalmtdTomato on the EV membranes, labelled EVs were dot blotted, which showed predominant PalmGFP/tdTomato signals associated with the inner membrane (Fig. 1g,h). Under this experimental set-up, these fusion proteins should only be readily detected in a concentration-dependent manner in the presence of a detergent (Tween-20), which opens EV membranes to allow antibody entry and labelling of the reporter proteins. In fact, only blots immunolabelled in the presence of the detergent revealed PalmGFP and PalmtdTomato signals when compared with the control (without detergent).

### PalmGFP/tdTomato label different sized EVs

**Figure 2:**
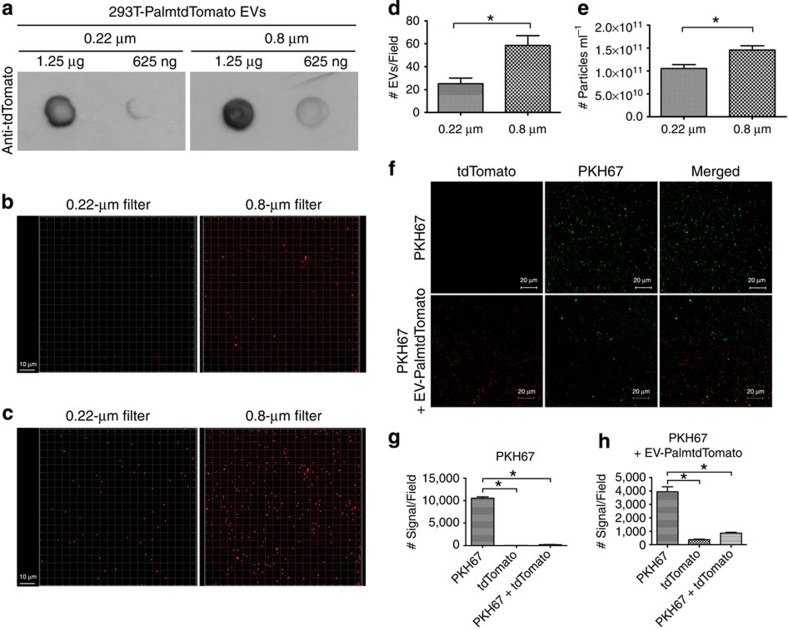
PalmtdTomato labels different-sized EVs and co-labels with PKH. (**a**) Dot blot detection of PalmtdTomato^+^ EV isolated from 0.22 or 0.8 μm filtered conditioned medium of 293T-PalmtdTomato cells. (**b**) Confocal microscopy of EVs isolated from 0.22 or 0.8 μm filtered conditioned medium of 293T-PalmtdTomato cells. Scale bar, 10 μm. (**c**) Digital display of PalmtdTomato^+^ EVs signals as spheres. Scale bar, 10 μm. (**d**) Quantification of PalmtdTomato^+^ EV signals per field in 0.22 or 0.8 μm filtered samples. *P<0.001 by two-tailed, *t*-test with 14 replicates. (**e**) Nanoparticle tracking analysis (NTA) of PalmtdTomato^+^ EVs isolated from 0.22 and 0.8 μm filtered samples. *P<0.05 by two-tailed *t*-test with six replicates. The results are presented as the mean ± s.e.m. (**f**) EV-depleted medium (top rows) or EVs isolated from 293T-PalmtdTomato cells (bottom rows) were stained with PKH67 followed by a 1 h wash in PBS at 100,000 *g*. Scale bar, 20 μm. (**g**,**h**) Semi-quantification of EV signals from PKH67-stained EV-depleted medium (**g**) or PalmtdTomato^+^ EVs co-labelling with PKH67 (**h**). *P<0.05 by repeated measures analysis of variance (ANOVA) followed by Tukey’s *post hoc* test with four replicates. The results are presented as the mean ± s.e.m.

To test whether the palmitoylated fluorescent EV reporters label more than one population of EVs, conditioned medium from 293T-PalmtdTomato cells was filtered through either a 0.22-μm filter or 0.8-μm filter before EV isolation. PalmtdTomato was readily detected in EVs isolated from both 0.22- and 0.8-μm filtered samples, indicating the reporter labels different sized EVs (Fig. 2a). Given the conventional dogma that exosomes range in size from 40 to 100 nm, while microvesicles fall between 100 and 1,000 nm in diameter^28^, the number of isolated EVs in the 0.22-μm filtered sample should be less than that in the 0.8-μm filtered sample. In fact, we did observe fewer EVs per field in the 0.22 μm sample as compared with the 0.8 μm sample, as assessed by semi-quantitative microscopic and quantitative nanoparticle tracking analysis of vesicle number (Fig. 2b–e).

### PalmtdTomato labels EVs more specifically than PKH67 dye

PKH fluorescent dyes, which label cell membranes by the insertion of their aliphatic chains into the lipid bilayer, have been widely used to label EVs based on their intense signal and long half-life^6^,^7^,^29^,^30^,^31^,^32^. However, we recently reported that the half-life of PKH dyes likely outlast that of EVs *in vivo,* thereby yielding inaccurate spatiotemporal assessments of EV fate^13^. We examined whether EV labelling with PKH dyes is entirely EV-specific and found that PKH67-labelled cell-free culture medium, initially used as a negative control, produced a higher signal than PKH67-labelled EVs isolated from 293T-PalmtdTomato cells under the same conditions (Fig. 2f–h). These results indicate that PKH67 not only labels EVs but may also aggregate and/or form micelles^33^, some of which are associated with pelleted EVs following an ultracentrifugation-based isolation procedure, resulting in false-positive signals for EVs. Although not statistically significant, we noted a small fraction of PKH67-labelled PalmtdTomato^+^ EV that are not detected with PKH67 alone (Fig. 2h), suggesting that it is PalmtdTomato protein that co-purified with EVs and/or there can be an artefact of Brownian motion, which produces a spatial shift between the tdTomato and PKH67 channels during image acquisition, thereby yielding non-PKH67 labelled PalmtdTomato^+^ signals.

### EV exchange between cell populations

**Figure 3:**
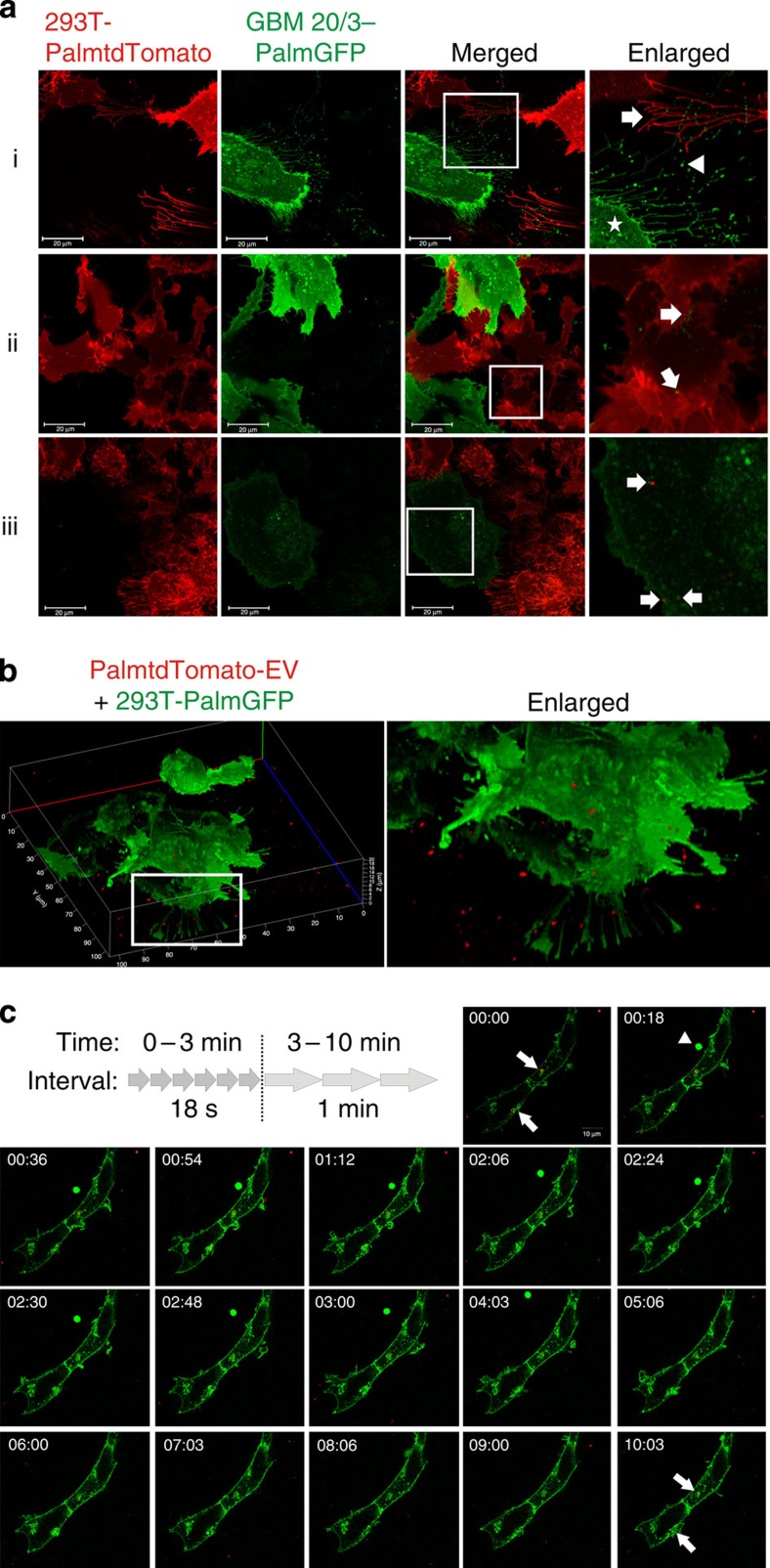
Live-cell imaging of EV exchange and uptake. (**a**) Stably transduced 293T-PalmtdTomato and primary GBM-PalmGFP cells were co-cultured and imaged with confocal microscopy for cellular interactions and EV exchanges between the cells. Magnification of boxes in merged panels are shown in far right panels. (i) Multiple thin cellular projections were readily observed extending from both GBM (arrowhead) and 293T (arrow) cells. Notably, many GBM cellular projections exhibited vesicle-like structure at the tips of the projections. Vesicle-like structures were also detected on the surface of GBM cells (star). (ii) PalmGFP^+^ EVs from GBM cells are found inside of 293T-PalmtdTomato cells (arrows). (iii) PalmtdTomato^+^ EVs from 293T cells are detected inside GBM-PalmGFP cells (arrows). Scale bar, 20 μm. (**b**) 3D reconstruction of Z-stack images showing 293T-PalmGFP cells exposed to PalmtdTomato^+^ EVs for 1.5 h. (**c**) Time-lapsed confocal imaging of PalmtdTomato^+^ EV attachment on 293T-PalmGFP cells at 2.45 h. Images shown at an 18-s interval from 0 to 3 min followed by a 1-minute interval from 3 to 10 min. PalmtdTomato^+^ EVs were surrounded by 293T-PalmGFP cell membranes (arrows) within a minute, and the pattern lasted until the end of the experiment (10 min). Notably, a round-shaped, PalmGFP-labelled entity with a diameter ≥ 4 μm (arrowhead) in the extracellular space was also observed from 00:18 to 04:03 min. Scale bar, 10 μm.

It is widely believed that EVs are dynamically exchanged between cell populations. However, most studies to-date, possibly due to a lack of a dual imaging system, have primarily focused on one-way delivery of EVs to cells. To demonstrate bi-directional EV exchange between cells without an intermediate purification step, we co-cultured 293T-PalmtdTomato and primary glioblastoma cells expressing PalmGFP (GBM-PalmGFP), and observed EV exchange between the differentially labelled cell types (Fig. 3a). Both cell types were also found to extend fine, long projections that can measure up to 60 μm in length and 200 nm in diameter, reminiscent of nanotubes^34^. Interestingly, some of the projections appeared to display EV-like structures at their tips.

### High-resolution tracking of EV uptake

To examine whether the fluorescent reporters can be used to track EVs at high-resolution, EVs isolated from 293T-PalmtdTomato cells were seeded onto 293T-PalmGFP recipient cells. Using live-cell confocal microscopy, PalmtdTomato^+^ EVs were detected around or on the 293T-PalmGFP cells (Fig. 3b), demonstrating that these reporters can also be used to distinguish recipient cells from the EVs. Further, time-lapse imaging of PalmtdTomato^+^ EV-exposed cells showed that EV attachment and uptake by recipient cells could be tracked in real-time using these reporters (Fig. 3c, Supplementary Movie 1, Supplementary Fig. 3). A PalmGFP vesicle-like signal with an estimated diameter of 4 μm near the recipient cells starting at 0:18 s was also observed. Given its relatively large size, it may represent cell debris and/or a type of oncosomes as previously described^35^, demonstrating that the fluorescent EV reporters can also label larger EVs. To further validate palmitoylated fluorescent EV uptake by the recipient cells, we demonstrated PalmtdTomato^+^ EV uptake by Gli36-PalmGFP glioma cells by fluorescence-activated cell sorting (FACS) analysis in the presence or absence of heparin, which reduced EV uptake as previously reported^36^ (Supplementary Fig. 4).

### Simultaneous visualization of EV and EV-packaged mRNA

**Figure 4:**
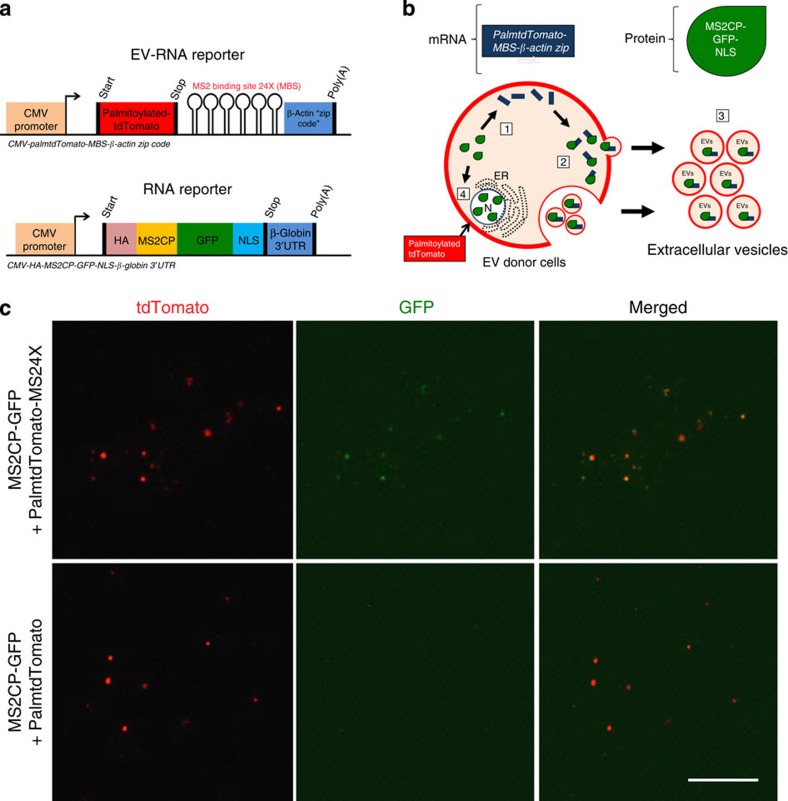
Visualization of EVs and packaged mRNAs. (**a**) Dual-function EV-RNA reporter system enabling EV and EV-RNA labelling; two constructs encoding either PalmtdTomato protein with RNA reporter transcripts containing repeat MS2 RNA binding sequences (MS24X; PalmtdTomato-MS24X; top), or MS2-GFP coat protein (MS2CP) with a nuclear localization signal (NLS) (bottom). (**b**) Diagram of vesicle RNA labelling process: [1] RNA reporter (blue bar) allows detection of EV-RNA reporter transcripts through binding of MS2 coat protein fused with GFP (MS2CP-GFP-NLS, green dewdrop) to PalmtdTomato-MS24X sequence in mRNA; [2] PalmtdTomato-MS24X:MS2CP-GFP RNA reporter complexes are packaged into EVs; [3] EVs are released from cells by budding from the cell membrane and/or fusion of multivesicular bodies with the cell membrane; and [4] nonspecific EV packaging of MS2CP-GFP-NLS is minimized by translocating the unbound RNA MS2CP-GFP reporter into the nucleus via the NLS signal. CMV, cytomegalovirus enhancer/promoter; HA, haemagglutinin tag; NLS, nuclear localization signal; UTR, untranslated region. (**c**) EVs were isolated from human Gli36 glioma cells expressing MS2CP-GFP with either PalmtdTomato-MS24X or PalmtdTomato alone (negative control for MS24X mRNA binding sites). Confocal microscopy imaging showed that only EVs isolated from cells co-expressing MS2CP-GFP and PalmtdTomato-MS24X exhibited co-localization between EVs (tdTomato) and MS2CP-GFP:MS24X mRNA complexes (GFP; *top row*). The negative control for MS24X (PalmtdTomato) showed only membrane labelled EVs (tdTomato). Scale bar, 10 μm.

Next, we designed a dual-function reporter for simultaneous visualization of both EV membranes and EV-RNAs (Fig. 4a). In this reporter scheme, PalmtdTomato protein was used for EV visualization while its transcript was tagged with a repeated MS2 RNA binding sequence in the 3′ UTR (PalmtdTomato-MS24X) for EV-RNA detection by bacteriophage MS2 coat protein fused with EGFP (MS2CP-GFP) expressed in the same cells (Fig. 4b). EVs isolated from Gli36 glioma cells co-expressing PalmtdTomato-MS24X and MS2CP-GFP exhibited clear co-localization between PalmtdTomato (EV) and MS2CP-GFP signals (PalmtdTomato RNA:MS2CP-GFP complex), whereas only PalmtdTomato (EV) but no MS2CP-GFP was detected in EVs from Gli36 expressing PalmtdTomato lacking the MS24X tag along with MS2CP-GFP (negative control; Fig. 4c). To further confirm that the co-localization of signals between PalmtdTomato and MS2CP-GFP is not a result of unbound MS2CP-GFP aggregates co-purified with PalmtdTomato^+^ EVs, the EV samples were imaged in the presence or absence of trypsin to remove EV surface bound proteins, and the co-localization was unaffected (Supplementary Fig. 5). To assess the diffraction limit of the microscope, we transfected siGLO RISC-free siRNA into 293T-PalmGFP cells and observed transfer of siRNA-packaged EVs to the Gli36 GBM-recipient cells with an estimated diffraction limit of 200–300 nm (Supplementary Fig. 6).

### Visualizing tumour-derived EVs *in vivo*

**Figure 5:**
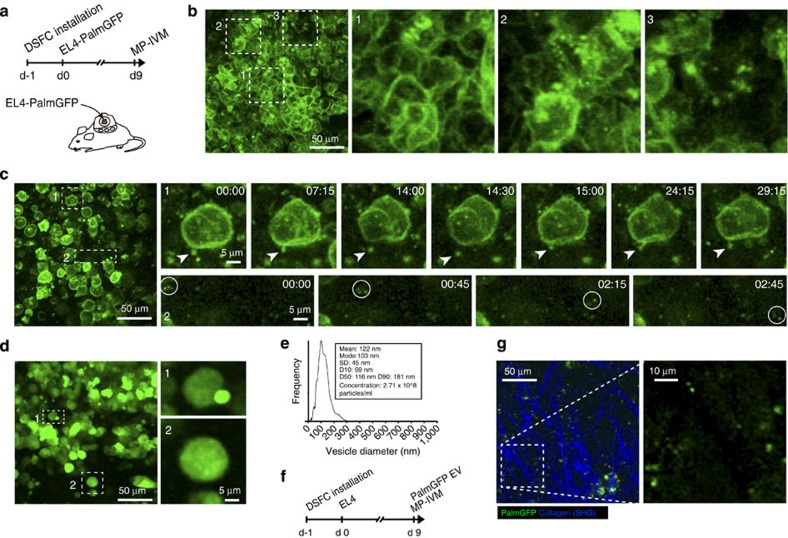
*In vivo* visualization of EVs. (**a**) PalmGFP-expressing EL4 tumours were implanted in pre-installed dorsal skinfold chambers (DSFCs) in C57BL/6 mice, and imaged 9 days later by MP-IVM. (**b**) Intravital micrograph from the central region of an EL4 tumour with regions characterized by the absence (subpanel 1), low (subpanel 2) and intermediate density (subpanel 3) of detectable PalmGFP^+^ vesicles. (**c**) Intravital micrograph of a peripheral region of a tumour with a high density of extracellular PalmGFP^+^ vesicles. Time-lapse recordings highlighting a tethered vesicle (subpanel 1) and a motile cluster of three vesicles (subpanel 2). Time is shown as min:s. (**d**) An identical experiment was performed as described for (**a**–**c**), but EL4 tumour cells expressing soluble GFP instead of PalmGFP were used instead. Note the absence of small micron- and submicron-sized particles (**e**) Nanoparticle tracking analysis of vesicles purified from the supernatant of PalmGFP-expressing EL4 tumour cells. (**f**) Non-fluorescent EL4 tumours were implanted into DSFCs and purified PalmGFP^+^ vesicles directly injected into the tumour on day 9. (**g**) Intravital micrograph of an EL4 tumour 60 min after injection of GFP^+^ EVs.

EV release and uptake are considered as potential mechanisms by which tumour cells communicate with each other and with their environment^2^. To test the general feasibility of visualizing EVs in tumour tissue by multiphoton intravital microscopy (MP-IVM), we stably expressed PalmGFP in the mouse thymoma cell line EL4 (EL4-PalmGFP) and implanted cells in dorsal skinfold chambers (DSFCs), where they formed solid tumours as described^37^,^38^ (Fig. 5a). When we imaged these tumours by MP-IVM 9 days later, we observed green fluorescent punctae that were either intracellular, associated with cytoplasmic membranes, or outside of EL4-PalmGFP tumour cells. The density of fluorescent punctae, which based on their origin likely represent different classes of tumour-derived vesicles, varied across the tumour environment. The lowest densities were typically observed in the areas of highest tumour cell density at the core of the tumour parenchyma (Fig. 5b, Supplementary Movie 2). More peripheral regions, in contrast, where the tumour parenchyma interfaces with the tumour stroma and where the density of tumour-infiltrating cells is lower while tumour-infiltrating immune cells, such as T cells and myeloid antigen-presenting cells, are more abundant^39^ showed the greatest density of fluorescent vesicles (Fig. 5c, Supplementary Movie 3). Time-lapse recordings showed that some of the larger EVs (estimated 1 μm diameter) often remained tethered to tumour cells for prolonged periods of time (Fig. 5c, subpanel 1, and Supplementary Movie 4). We did not observe this behaviour for smaller EVs, which may be due to the enhanced difficulty of tracking these by imaging of thin optical sections in the three-dimensional environment of tumours *in vivo* (as opposed to the two-dimensional environment of a culture dish). However, both small and larger EVs were observed to traffic individually within the tissue or to group in small, fast-moving clusters, suggesting that they were attached to or had been internalized into a motile, non-visualized cell population, such as tumour-infiltrating immune cells (Fig. 5c, subpanel 2, and Supplementary Movie 5).

EL-4 tumours expressing soluble instead of membrane-targeted GFP also produced a small number of fluorescent objects of subcellular size *in vivo*. However, these objects, which likely represent tumour cell apoptotic bodies, were much larger (up to 5 μm in diameter) and less numerous than the small membrane-derived fluorescent punctae observed in EL4-Palm-GFP tumours (Fig. 5d, Supplementary Movie 6 and 7), indicating that the latter are indeed tumour membrane-derived vesicles, and not simply tumour cell debris that has been ingested by non-visualized phagocytes.

To assess whether the EVs detectable in EL4-PalmGFP tumours are likely to correspond to EVs as characterized *in vitro*, we purified EVs of sizes up to 300 nm from these tumour cells *in vitro* (Fig. 5e) and injected these into established, non-fluorescent EL4 tumours in DSFCs. One hour later we observed, amid the anatomical landmark structures of tumoral collagen fibres, Palm GFP^+^ EVs with a similar size distribution as observed in EL4-PalmGFP tumours (Fig. 5f). While our imaging system is likely not sensitive enough to detect the smallest EVs, this suggests that EVs rapidly coalesce in tumour tissue, probably in part through uptake by non-tumour cells. Collectively, these observations establish the feasibility of investigating the dynamic behaviour of tumour-derived EVs *in vivo* through fluorescent protein-labelling of tumour cell membranes.

### EV uptake and nascent EV-RNA translation

**Figure 6:**
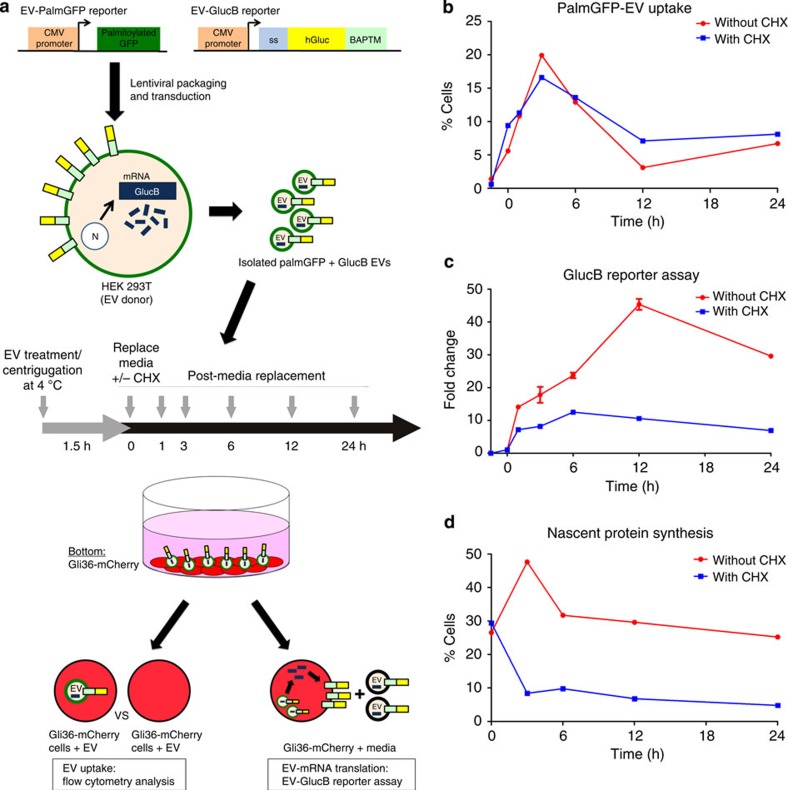
EV uptake and EV-RNA translation. (**a**) Schematic of two expression reporters used to detect EV uptake (PalmGFP) and translation of EV-delivered GlucB mRNA (luciferase activity). 293T cells (donors) were stably transduced with lentivirus vectors expressing these reporters. EVs were isolated from conditioned medium of these cells and added to Gli36-mCherry glioma cells followed by centrifugation at 4 °C to facilitate EV docking on cells while minimizing EV uptake. PalmGFP^+^GlucB^+^ EV-containing media was then replaced with EV-depleted media in the presence and absence of cycloheximide (CHX) and cells were incubated at 37 °C for 24 h. Medium and cell samples were collected at indicated time points to assess EV uptake (fluorescence and luciferase activity) and EV-mRNA translation (luciferase activity). CMV, cytomegalovirus enhancer/promoter; ss, signal sequence; BAPTM, biotin acceptor peptide with transmembrane domain of platelet-derived growth factor receptor. (**b**) Flow cytometry analysis of the percentage of Gli36-mCherry cells positive for PalmGFP^+^ EV uptake in the presence or absence of CHX. Cells that have taken up EVs exhibited signal for both PalmGFP (EVs) and mCherry (recipient cells), whereas cells without or with low levels of EVs showed only mCherry (see also Supplementary Fig. 5). (**c**) GlucB reporter assay was performed to detect translation of EV-delivered GlucB mRNA by Gli36-mCherry cells expressed as fold change. (**d**) Flow cytometry analysis of nascent protein synthesis in Gli36-mCherry cells revealed by Click-iT HPG labelling and Alexa Fluor488 conjugation expressed as percentage of cells positive for Alexa Fluor 488 (see also Supplementary Fig. 6).

While EVs have been shown to mediate RNA and protein transfer to elicit phenotypic changes in recipient cells^5^, detailed temporal properties of these events are still under study. We multiplexed the fluorescent (PalmtdTomato) and bioluminescent (EV-GlucB)^13^ EV membrane reporters monitor the time of EV uptake and EV-mRNA translation in the recipient cells (Fig. 6a). Using centrifugation at 4 °C to promote EV uptake and restrict the time window of uptake followed by FACS analysis, we determined that EVs from 293T-PalmGFP+GlucB cells were actively taken up by the recipient Gli36-mCherry glioma cells, between 0 and 3 h following EV treatment (Fig. 6b; Supplementary Fig. 7). This upward trend was followed by a decrease in EV signal from 3 to 12 h, suggesting that EVs and associated proteins are processed and degraded during this time. Interestingly, the signal came to a plateau from 12 to 24 h, but did not reach the baseline, implying that (i) a small proportion of EVs may remain intact in the recipient cells and/or (ii) the EVs were not fully degraded and some EV membrane (with the PalmGFP reporter) remained in the cells. Treatment with the protein translation inhibitor, cycloheximide (CHX), did not appear to affect EV uptake and dynamics in the recipient cells (Fig. 6b).

To detect and monitor translation of EV-delivered mRNA in parallel with EV uptake, EV-treated cells and conditioned media were collected and luciferase activity of the GlucB reporter was measured. If the recipient cells translate EV-delivered GlucB mRNA, an increase in GlucB signal should be observed, whereas treatment with CHX should prevent GlucB mRNA translation and reduce any increase in the signal. Remarkably, the elevation in GlucB signal was observed as early as 1 h post-EV exposure, indicating that translation of EV-delivered GlucB mRNA began soon after EV uptake by the cells (Fig. 6c). The increase continued and reached a peak at 12 h and was followed by a decline between 12 and 24 h. This pattern directly correlated with levels of EV uptake (Fig. 6b) where highest and lowest percentage of EV-containing cells were observed between 0–12 h and 12–24 h, respectively. In addition, samples treated with CHX consistently showed a significantly lower level of GlucB signal when compared with non-CHX-treated samples, with these translation-blocked samples serving as a baseline reading for GlucB reporter protein directly transferred by the EVs. Furthermore, nascent protein synthesis was confirmed to be suppressed by CHX treatment throughout the experiment as revealed by Click-iT homopropargylglycine (HPG) labelling and FACS analysis (Fig. 6d; Supplementary Fig. 8). We thereby successfully monitored and revealed temporal dynamics of both EV uptake and translation of EV-delivered mRNA.

Next we wanted to further confirm that RNA transfer by EVs could indeed induce a physiological response in recipient cells. Given the pivotal role of the nuclear factor kappa B (NFκB) in different physiological processes and the fact that its regulation can be modulated through various exogenous factors^40^, we investigated NFkB activity following EV-mediated RNA transfer. 293T recipient cells were engineered to stably express a bioluminescent NFkB reporter driving the expression of Gluc (NFkB-Gluc)^41^,^42^ and treated with 293T-EVs. We observed a significant elevation in NFkB activity from 24 to 72 h followed by a decline at 96 h post-EV treatment (Supplementary Fig. 9a,b). As EVs contain lipids, proteins and other nucleic acids in addition to RNA, the observed increase in NFkB activity could be attributed to multiple factors. To distinguish whether this effect on NFkB is, at least in part, contributed by EV-delivered RNA, the recipient cells were transfected with varying amount of RNA isolated from the EVs and showed a dose-dependent rise in NFkB activity from 24 to 72 h post-EV-RNA transfection, corroborating the trend of EV-induced NFkB activation (Supplementary Fig. 9c). While it remains to be determined which RNAs resulted in the current observation, we hereby demonstrated that the delivery of EV-RNA alone could trigger cell signalling and induce an NFkB response over an extended period of time in recipient cells.

## Discussion

Given that most EVs are nanosized as opposed to micrometer-sized cells, an ideal EV labelling method for imaging at subcellular resolution should meet the following criteria: (i) specific labelling of EVs; (ii) stable and sufficient signal to detect nanoscaled EVs from background noise; and (iii) a half-life consistent with that of EVs. The palmitoylated (Palm) fluorescent EV reporters described here achieved these needs for EV visualization, enabling (a) labelling of multiple EV types irrespective of their biogenesis; (b) semi-quantification of EVs; (c) time-lapse live-cell imaging of EV release and uptake; (d) EV exchange between different cell populations; (e) intravital EV imaging; and (f) FACS analysis of EV uptake.

PalmGFP and PalmtdTomato, in contrast to labelling with EV-enriched protein conjugated fluorescent proteins such as CD63-GFP^43^, are designed to be a general EV labelling strategy to visualize and track multiple EV subtypes. This was shown by detection of PalmGFP^+^ and PalmtdTomato^+^ EVs in both 0.22- and 0.8-μm sized EV populations, as well as sucrose density gradient with EV-marker proteins, such as Alix. Interestingly, using these reporters, we also observed EV-like structures at the tips of cell projections. As nanofilaments were recently found to be present on glioblastoma exosomes using peak force microscopy^44^, these cell projections with EV-like structures may represent another mechanism by which EVs are released in addition to the previously described plasma membrane budding of microvesicles and exosome release from the multivesicular bodies. By labelling different cell populations in co-culture, it was also possible to monitor the exchange of EVs between cell types, demonstrating that EV transfer is dynamic and bidirectional.

PKH, along with other lipophilic dyes such as DiI, represents one of the most commonly used dyes to label EVs^6^,^7^,^32^. However, here we showed that PKH67 staining of EVs is not EV-specific, and that it labels other lipid-containing entities in the extracellular space, and presumably in recipient cells, potentially leading to false-positive signals. Furthermore, PKH dyes were reported to have an *in vivo* half-life of 5 to >100 days^45^. While its long half-life may be advantageous for some purposes, the dye may outlast labelled EVs following EV processing/degradation by cells, leading to misinterpretation of dye residues and/or recycled dye-labelled membrane as originally labelled EVs. In fact, our recent study showed that intravenously administered EVs have an *in vivo* half-life of <30 min in most tissues^13^. Using PalmGFP^+^ EVs, we showed that the *in vitro* half-life of EVs in recipient cells is <24 h, which implies that any vesicle-like structures labelled with PKH dyes after this time are unlikely to be EVs. In addition, as EV surface proteins, at least in part, have been reported to be responsible for the binding and subsequent uptake of EVs by the recipient cells^46^,^47^, it is ideal to minimize perturbation to the EV surface proteome during labelling. Here we demonstrated that both PalmGFP and PalmtdTomato predominantly label the inner EV membrane, thereby reducing potential disturbance to the EV surface protein composition.

We also confirm here, using direct *in vivo* visualization through MP-IVM, that tumour cells release EVs of varying sizes, at least some of which are likely taken up by migratory, non-tumour cells, likely migratory immune cells. Simultaneous visualization of different immune cell subsets in future studies will provide clues to reveal the identity of the cell types actively taking up EVs released by tumour cells. Implantation of mixtures of tumour cell clones expressing fluorescent proteins in complementary colours will also allow us to monitor EV exchange between tumour cells, which may be masked in our current system. Interestingly, EV density was highest at the tumour-parenchyma border, suggesting that EV formation or release may be enhanced as a result of the interaction of malignant cells with the surrounding normal tissue microenvironment, including the immune cells.

We witnessed both endogenously generated and exogenous RNA cargos of EVs using the MS24X RNA sequence in combination with MS2CP-GFP in the donor cells^48^ or by transfection of donor cells with fluorescently labelled siRNA, respectively. We further investigated nascent translation of EV RNA cargo and revealed translation of EV-mRNA within 1 h following EV uptake by recipient cells. This timeframe coincides with that of liposome-delivered mRNAs where translation occurs within 1 h post-transfection^49^. Interestingly, the decrease in GlucB signal from 12 to 24 h corresponds to a reduction in the percentage of cells showing labelled EVs present during this same time period, suggesting that the decreased GlucB signal is a result of decreasing availability of external EVs combined with internal degradation of GlucB transcripts.

EV uptake has been shown to occur via multiple routes, including a direct fusion between EVs and the plasma membrane^50^, as well as EV internalization via lipid raft-, clathrin- and calveolae-dependent endocytosis, macropinocytosis and phagocytosis^47^,^51^,^52^,^53^,^54^,^55^. However, it remains to be determined as to which EV uptake mechanism(s) is employed in different cell types under various conditions. The route of EV uptake is likely dependent on the following factors: (a) lipid and protein composition of the EV and plasma membranes of donor and recipient cell types; (b) EV subtypes; (c) state of cells releasing and taking up EVs, for example, healthy or diseased; and (d) conditions in the extracellular space, for example, acidic versus basic, hypoxic, extracellular matrix components and so on. The study presented here provides a means of successfully monitored EV docking and uptake, which will allow elucidation of these release and uptake mechanisms. It should also be noted that inhibition of protein translation in recipient cells by CHX treatment did not appear to affect EV uptake in this experimental set-up. We speculate this could be a result of (1) uniform attachment of EVs by centrifugation at 4 °C before the CHX treatment; (2) pre-existing proteins available for the subsequently internalization of EVs; and (3) docked EV-cell membrane fusion that can readily occur when the temperature resumed to 37 °C following the centrifugation. Future studies will provide insight as to whether nascent protein synthesis is required for EV uptake via endocytosis and/or membrane fusion.

We further demonstrated that, under our experimental set-up, EV-mediated RNA delivery can induce NFkB activity over an extended period of time (up to 72 h) in the recipient cells in a dose-dependent manner. EVs from normal and tumour cells are capable of modulating the immune response, as well as the cellular physiology in the tumour microenvironment^56^,^57^,^58^,^59^. Moreover, here we show direct evidence that EV-RNA can affect major signalling pathways in recipient cells such as NFkB, a key mediator of the immune response as well as inflammation and tumorigenesis^60^. While the physiological relevant EV dose and treatment duration remainns to be defined, the current findings suggest that uptake of EVs by recipient cells in a microenvironment and at distal sites under pathological conditions, such as cancer, could result in immediate and systemic NFkB responses, respectively.

Our findings support EV-mediated communication between cells, which can be complex, multi-directional and far-reaching. Protein and RNA cargos can not only be transferred to elicit phenotypic changes in recipient cells, but foreign mRNAs contained within EVs can also be rapidly translated. This latter finding supports potential RNA therapeutic applications of vesicles^5^. Future studies should focus on the dynamics of EV biogenesis, release and uptake considering EVs as a multifaceted communication platform between local and distant cell populations.

## Methods

### Cell culture

Human embryonic kidney 293T cells (American Type Culture Collection (ATCC), Manassas, VA), Gli36 glioma cells (Dr Anthony Capanogni, UCLA, Los Angeles, CA) and primary GBM cells 20/3^7^ were cultured in high-glucose Dulbecco’s modified Eagle’s medium (Corning Cellgro, Manassas, VA) containing 10% fetal bovine serum (FBS) (Sigma, St Louis, MO) and 100 U ml^−1^ penicillin, 100 μg ml^−1^ streptomycin (Invitrogen, Grand Island, NY) in a humidified atmosphere at 37 °C, 5% CO_2_. EL4 cells (ATCC) were maintained in RPMI 1640 adjusted to contain 1.5 g l^−1^ sodium bicarbonate, 4.5 g l^−1^ glucose, 10 mM HEPES, 1.0 mM sodium pyruvate and supplemented with 0.05 mM 2-mercaptoethanol and 10% fetal bovine serum.

### Reporter constructs

Palmitoylation sequences (MLCCMRRTKQ) of growth cone-associated protein (GAP43)^61^ were genetically fused to the NH_2_ terminus of GFP (PalmGFP) and tdTomato (PalmtdTomato) by PCR using Phusion High-Fidelity DNA Polymerase (New England BioLabs, Ipswich, MA, USA). Plasmids pCAG-mGFP (Addgene plasmid 14757) (ref. 62) and pCSCMV:tdTomato (Addgene plasmid 30530) (ref. 63) were used as cDNA templates for GAP-43, EGFP and tdTomato (Supplementary Table 1 for primers). PalmGFP and PalmtdTomato sequences were inserted at NheI and XhoI sites of CSCGW2 lentivector plasmid kindly provided by Dr Miguel Sena-Esteves (University of Massachusetts Medical School, Worcester, MA, USA)^64^, tagged with MS24X repeats between PspOMI and HpaI sites followed by β-actin 3′ UTR (a kind gift from Dr Robert Singer, Albert Einstein College of Medicine, Bronx, NY, USA) between HpaI and XhoI sites. EV-GlucB construct was used to label EVs with a membrane-bound version of Gluc as previously described^13^.

### EV production and isolation

Cells were transfected with DNA constructs using polyethyleneimine^65^, siGLO RISC-free siRNA with Lipofectamine 2,000 (Invitrogen), or stably transduced with packaged lentivirus vectors (MGH Vector Core, Boston, MA, USA) to express genes of interest. EVs were isolated, as previously described^13^. In brief, conditioned medium was collected from cells incubated with culture medium supplemented with 10% EV-depleted FBS for 48 h, centrifuged at 300 *g* for 10 min, 2,000 *g* for 10 min at 4 °C, and filtered through a 0.8 μm (or 0.22 μm as specified) filter (Millipore, Billerica, MA, USA) before ultracentrifugation at 100,000 *g* for 90 min at 4 °C. EV pellets were resuspended with double-0.22 μm-membrane filtered PBS.

### Live-cell confocal microscopy

Micrographs were captured with an LSM510 confocal microscope equipped with either 63 × Zeiss Plan-APOCHROMAT oil, 1.4NA or 100 × Zeiss Plan-APOCHROMAT DIC oil, 1.46NA under environmental control at 37 °C and 5% CO_2_ (Zeiss, Thornwood, NY, USA).

### EV semi-quantification

Isolated EVs were first mounted onto a microscope slide with a #1 circular coverglass, allowed to settle for 30 min at room temperature and imaged by confocal microscopy. To semi-quantitate EVs, EV signals in the confocal micrographs were first digitally displayed as spheres and then semi-quantitated by Imaris software (Bitplane, South Windsor, CT, USA). Same threshold for EV digital display and semi-quantification was applied to all samples. For semi-quantification of Palmtdtomato-EV and PKH67 signals, confocal micrographs were analysed by using MCID software (MCID Image Analysis Software Solutions for Life Sciences, USA).

### Sucrose density gradients and western blot analysis

Isolated EVs were subjected to sucrose density gradients consisting of 8, 30, 45 and 60% layers in PBS and centrifuged at 232,500 *g* for 30 min at 4 °C. Following removal of the top layer, 10 fractions were collected and diluted 1:10 in PBS followed by centrifugation at 100,000 *g* to collect EV-containing pellets.

The pellets were lysed with 25 μl RIPA buffer supplemented with protease inhibitor (mini, Complete, Roche Diagnostics, Indianapolis, IN, USA), resolved by 10% SDS–PAGE with molecular weight standards (Precision Plus Protein All Blue Standards, Bio-Rad, Hercules, CA, USA) and transferred onto nitrocellulose membranes. The membranes were blocked with 5% bovine serum albumin (BSA) fraction V (Invitrogen), immunoblotted with anti-GFP (mouse; Invitrogen), anti-dsRed (to detect tdTomato; rabbit; Clontech, Mountain View, CA, USA) and anti-Alix (mouse; Santa Cruz, Dallas, TX, USA) antibodies followed by secondary antibodies conjugated to horseradish peroxidase (HRP; Molecular Probes, Eugene, OR, USA). Signal was detected with SuperSignal West Pico Chemiluminescent Substrate (Thermo Scientific, Rockford, IL, USA).

### Transmission electron microscopy and immunogold staining

Isolated EVs were pelleted at 20,000 g for 30 min at 4 °C, fixed with 4% formaldehyde in PBS for 2 h, washed in PBS and embedded in 20% gelatin. The gelatin embedded pellet was infiltrated in 2.3 M sucrose (cryoprotection) and frozen in liquid nitrogen (LN2). Ultrathin (approximately 80 nm) frozen sections were cut at −120 °C and picked up on formvar/carbon-coated copper grids. The thawed sections were immunolabelled with anti-GFP (rabbit, AbCam #6556) or anti-dsRed (mouse; Clontech) followed by 5 or 10 nm protein A-gold secondary antibodies (University Medical Center, Utrecht, The Netherlands), respectively. Images were captured using a Tecnai G2 Spirit Bio TWIN transmission electron microscope.

### Multiphoton intravital microscopy (MP-IVM) of mouse tumours

DSFCs were installed on C57BL6 (B6) female mice (The Jackson Laboratory, Bar Harbor, ME), as previously described^37^,^38^. One day later, 10^6^ EL4, EL4-PalmGFP or EL4-GFP tumour cells in 50 μl HBSS were implanted into the chambers by injection into the skin tissue. Nine days later, animals were anaesthetized by intraperitoneal injection of Ketamine and Xylazine, and positioned on a custom-made microscopy stage that permitted maintaining the temperature of exposed skin tissue at 37 °C. Purified EVs from EL4-PalmGFP cells were injected into tumours through the intact skin from the side opposing the exposed skin surface. All experiments were in accordance with NIH guidelines and were approved by the Institutional Animal Committees of Massachusetts General Hospital.

### EV uptake analysis and EV-RNA translation assay

For the transwell set-up to assess the diffraction limit and sensitivity of the microscope on EVs, cell culture inserts with 1 μm pore (BD Biosciences, San Jose, CA, USA) in a 24-well plate placed with coverslips within the wells were used.

For EV donors, 293T cells were infected with lentiviruses to stably both express PalmGFP and GlucB reporters^13^. PalmGFP+GlucB^+^ EVs were isolated from 0.8-μm filtered conditioned medium of the transduced cells, as described above. For EV recipient cells, Gli36 cells were stably transfected to express mCherry reporter (Gli36-mCherry).

To allow uniform docking of EVs and halt EV uptake by the recipient cells, Gli36-mCherry cells in a 24-well plate were treated with PalmGFP+GlucB^+^ EVs in EV-depleted medium and centrifuged at 1,800 r.p.m. (maximum rotor radius: 19.2 cm) at 4 °C for 90 min. PalmGFP+GlucB^+^ EV-containing medium was then removed, and the cells were washed once with PBS followed by replacement with EV-depleted medium with or without cycloheximide (CHX; 20 μg ml^−1^). At the time of centrifugation (−1.5 h), 0, 1, 3, 6, 12 and 24 h post-centrifugation, conditioned medium was first collected, and cells were washed with PBS followed by trypsinization with 0.05% trypsin/0.53 mM EDTA (Corning Cellgro) and resuspension in PBS for flow cytometry analysis on a BD LSRII Multi-Laser Analyser (BD Biosciences) to determine EV uptake by the recipient cells.

To assess translation of EV-delivered GlucB RNA, the collected cells and conditioned medium were plated in triplicates into a white 96-well luminometer plate. GlucB activity was then measured by an MLX Microtiter plate luminometer (Dynex Technologies, Chantilly, VA, USA) with automated injection of 50 μl coelentrazine (CTZ; 8 ng ml^−1^; Nanolight, Pinetop, AZ).

### Nascent protein synthesis assay

To detect CHX-mediated inhibition of protein synthesis, Gli36-mCherry cells in a 24-well plate were prepared in parallel to the EV uptake and EV-RNA translation experiments as described above. At the indicated post-centrifugation time points, culture medium was replaced with methionine- and cysteine-free medium containing Click-iT HPG (Invitrogen) for 30 min, and the cells were washed with PBS, trypsinized with 0.05% trypsin/0.53 mM EDTA (Corning Cellgro), resuspended in PBS, pelleted at 1,500 r.p.m. (maximum rotor radius: 20.78 cm) for 5 min at 4 °C and resuspended in 80% ethanol for 15 min on ice. The fixed cells were conjugated with Alexa Fluor 488 azide to reveal HPG labelling, and stained with NuclearMask Blue stain according to the manufacturer’s protocol (Invitrogen). The labelled cells were resuspended in PBS for flow cytometry analysis on a BD LSRII Multi-Laser Analyser (BD Biosciences).

## Additional information

**How to cite this article:** Lai, C.P. *et al.* Visualization and tracking of tumour extracellular vesicle delivery and RNA translation using multiplexed reporters. *Nat. Commun.* 6:7029 doi: 10.1038/ncomms8029 (2015).

## Supplementary Material

Supplementary Figures Supplementary Figures 1-9 and Supplementary Table 1

Supplementary Movie 1 PalmtdTomato^+^ EV docking/uptake by 293T-PalmGFP cells. A live-cell confocal microscopy recording at 2.45 h post-PalmtdTomato^+^ EV treatment on 293T-PalmGFP cells. Scale bar = 20 μm. Time is shown in minutes and seconds.

Supplementary Movie 2 Core of EL4-PalmGFP tumor parenchyma. An MP-IVM recording from an EL4-PalmGFP tumor implanted into a dorsal skinfold chamber 9 days earlier. Note the paucity of GFP^+^ vesicles in most areas. Few tumor cells exhibit intracellular vesicle clusters. Each individual frame is a maximum intensity projection of 11 z-stacks spaced 4 μm apart (total thickness of 40 μm). Scale bar = 50 μm. Time is shown in minutes and seconds.

Supplementary Movie 3 Parenchyma/stroma interface of EL4-PalmGFP tumor. An MP-IVM recording from an EL4-PalmGFP tumor implanted into a dorsal skinfold chamber 9 days later. Note the much greater density and motility of extracellular GFP+ vesicles compared to Supplementary Video 2. Also, more tumor cells exhibit intracellular vesicle clusters. Each individual frame is a maximum intensity projection of 11 z-stacks spaced 4 μm apart (total thickness of 40 μm). Scale bar = 50 μm. Time is shown in minutes and seconds.

Supplementary Movie 4 Stably tethered tumor cell vesicle. A magnified view of a section of Supplementary Video 3. The circle highlights a large vesicle that remains tether to a tumor cells for the duration of the recording. Each individual frame is a maximum intensity projection of 11 z-stacks spaced 4 μm apart (total thickness of 40μm). Scale bar = 5 μm. Time is shown in minutes and seconds.

Supplementary Movie 5 Tumor cell vesicles moving in clusters. A magnified view of a different section of Supplementary Video 3. The circle appearing at time-point 14 min 15 sec highlights a group of vesicles that rapidly traverse the field of view, suggesting that they have been taken up by the same motile cell. Each individual frame is a maximum intensity projection of 11 z-stacks spaced 4 μm apart (total thickness of 40μm). Scale bar = 5 μm. Time is shown in minutes and seconds.

Supplementary Movie 6 Core of EL4-GFP tumor parenchyma. An MP-IVM recording from an EL4 tumor expressing soluble GFP and implanted into a dorsal skinfold chamber 9 days earlier. Note the absence of GFP^+^ vesicles. Each individual frame is a maximum intensity projection of 11 z-stacks spaced 4 μm apart (total thickness of 40 μm). Scale bar = 50 μm. Time is shown in minutes and seconds.

Supplementary Movie 7 Parenchyma/stroma interface of EL4-GFP tumor. An MP-IVM recording from an EL4 tumor expressing soluble GFP and implanted into a dorsal skinfold chamber 9 days earlier. Note the absence of GFP+ vesicles. Each individual frame is a maximum intensity projection of 11 z-stacks spaced 4 μm apart (total thickness of 40 μm). Scale bar = 50 μm. Time is shown in minutes and seconds.
