# Supplementary material for: Visualization and tracking of tumour extracellular vesicle delivery and RNA translation using multiplexed reporters
Source: Nat Commun. 2015 May 13;6:7029. doi: 10.1038/ncomms8029 (PMC4435621; doi:10.1038/ncomms8029)
Supplement: Supplementary Figures — 1-9 and Supplementary Table 1 [file ncomms8029-s1.pdf]

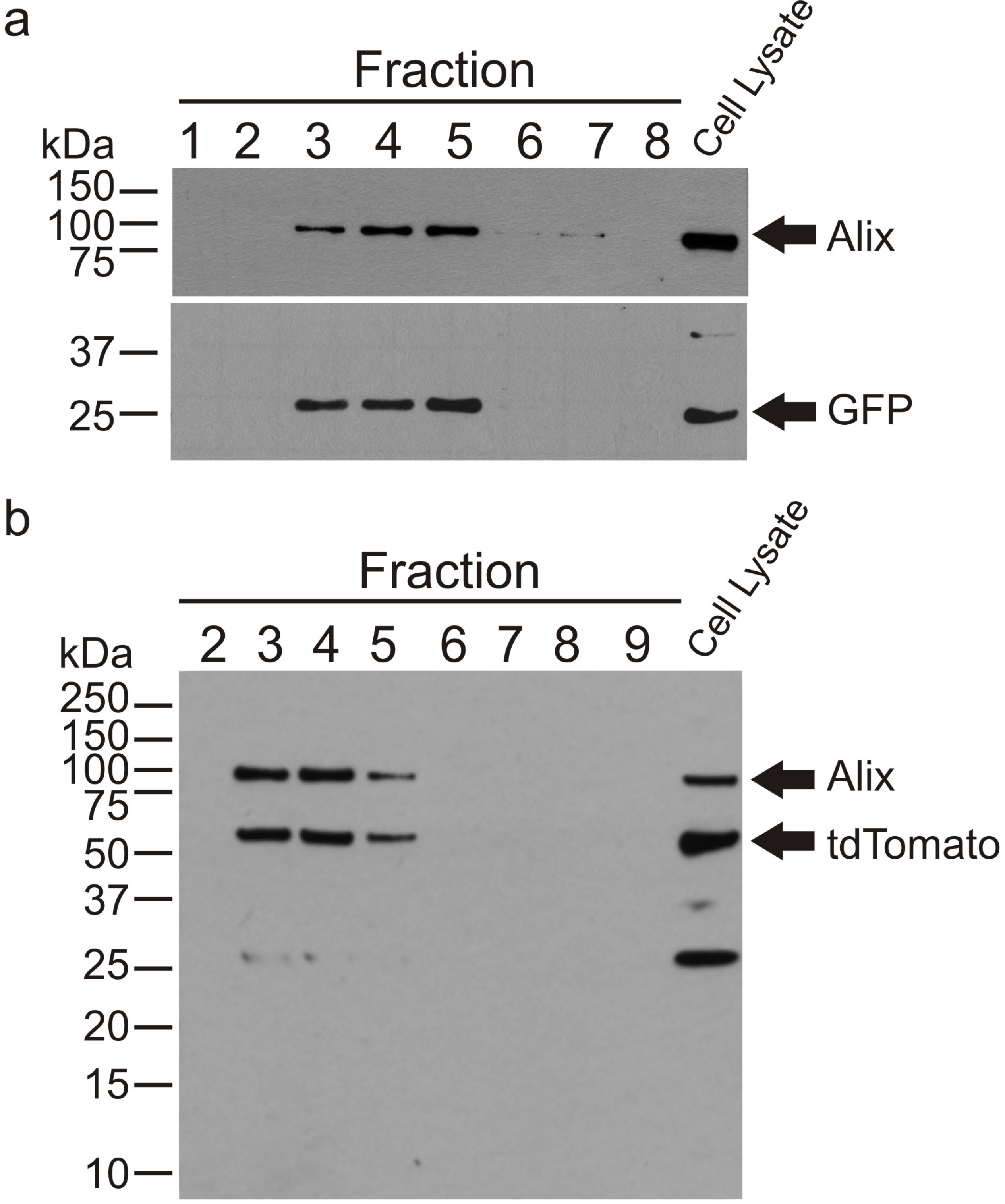

Supplementary Figure 1. Western blots of Fig. 1c, d. (**a, b**) Western blot analysis of pelleted proteins from fractions collected following sucrose gradient centrifugation of PalmGFP- (**a**) and Palmtdtomato-labeled EVs (**b**). Cells lysate of 293-PalmGFP or -PalmtdTomato were used as a positive control. Immunoblotting with anti-tdTomato antibody revealed a band at 25 kDa predominantly in the cell lysate but not in the EV-containing fractions, indicating it may be cleaved monomers of tdTomato.

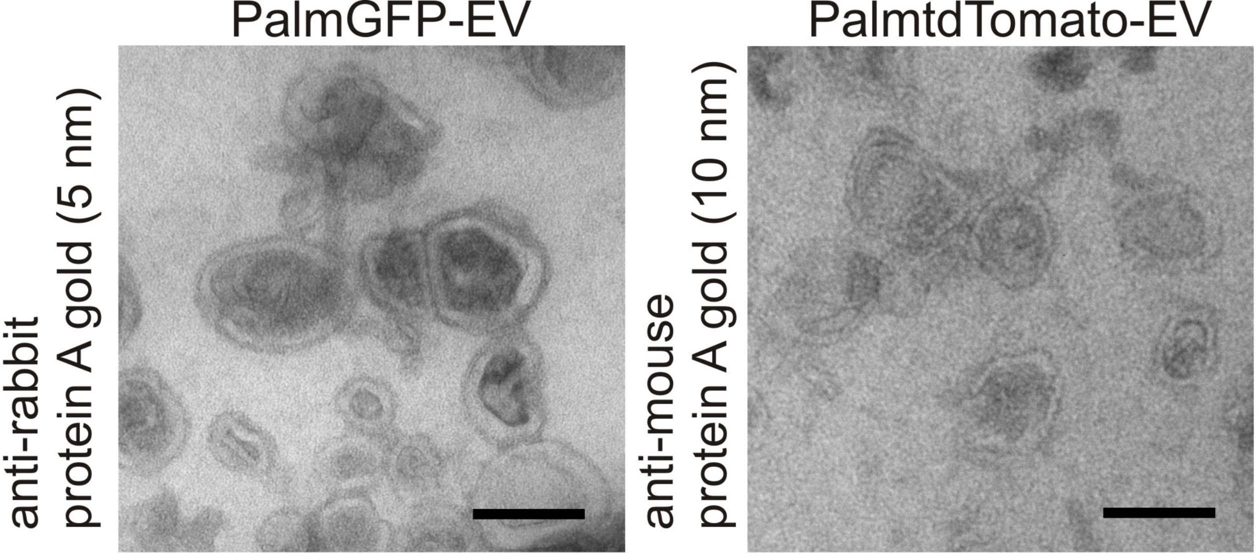

Supplementary Figure 2. Secondary antibody control immunolabeling of PalmGFP- and PalmtdTomato-EVs. PalmGFP- and PalmtdTomato-EVs were immunolabeled with anti-mouse protein A gold (5 nm) and anti-rabbit protein A gold (10 nm) antibodies, respectively. TEM shows no non-specific binding of the secondary antibodies to PalmGFP (left) and PalmtdTomato-EVs (right). Bar, 100 nm.

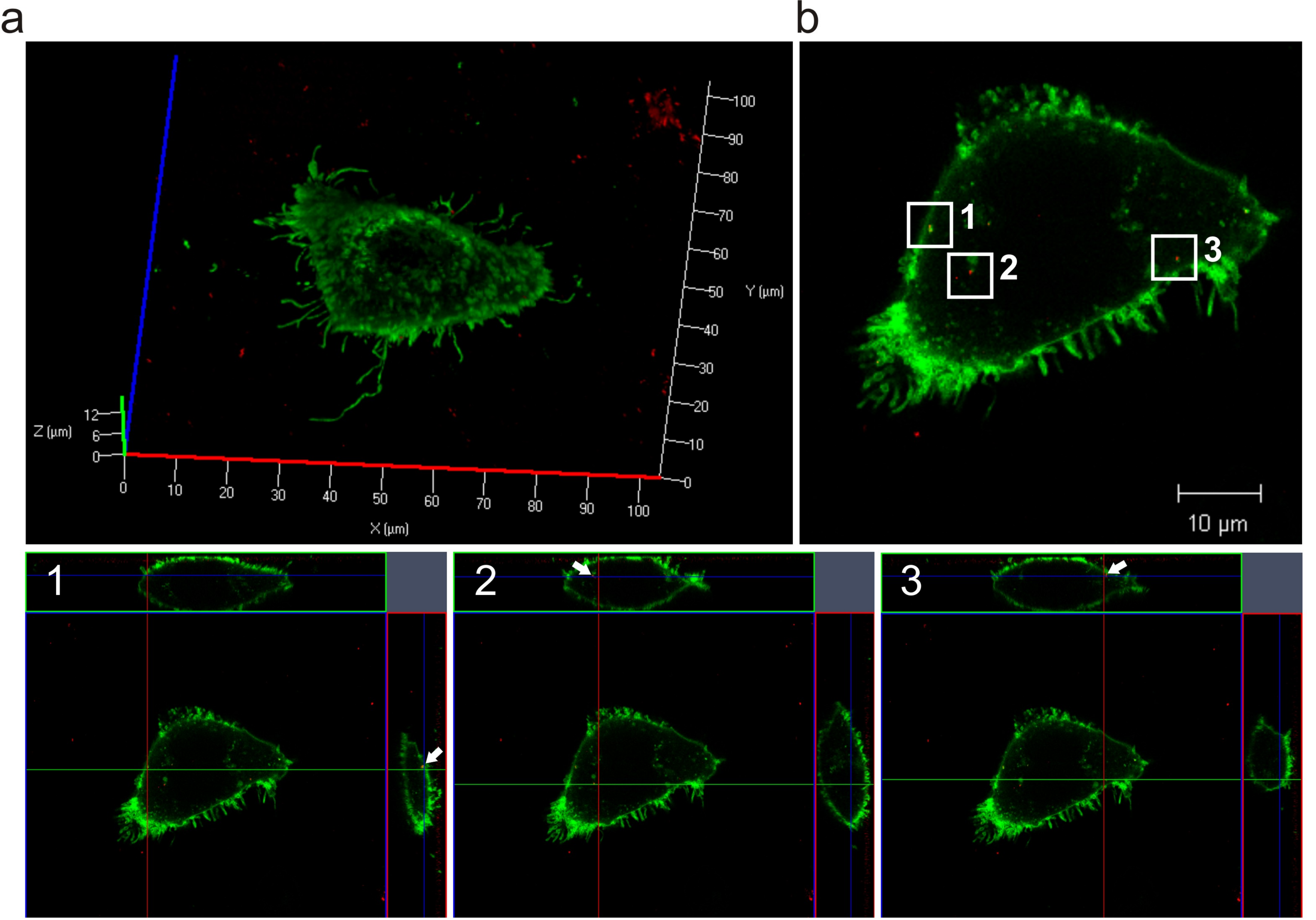

Supplementary Figure 3. EV docking and internalization by recipient cells. (**a**) 3D reconstruction of Z-stack images showing 293T-PalmGFP cells exposed to 293T-PalmtdTomato^{+} EVs for 1.75 h. (**b**) Single Z-plane confocal image and orthogonal projections from Z-stack images (subpanel 1-3) demonstrating EV docking on the plasma membrane (arrow; subpanel 1 and 3) and uptake by the recipient cell (arrow, subpanel 3). Bar, 10 µm.

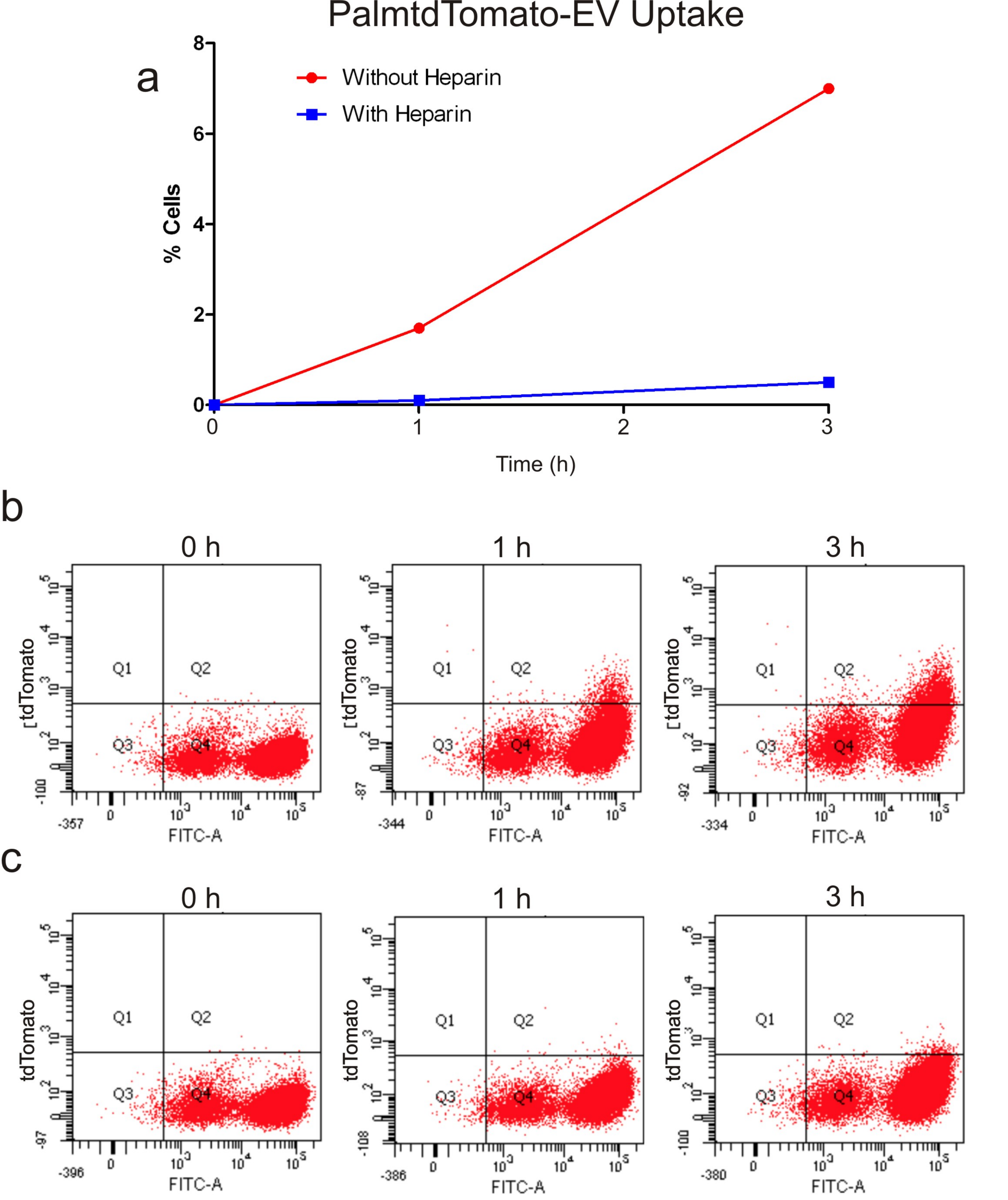

Supplementary Figure 4. Heparin-mediated inhibition of 293T-PalmtdTomato⁺ EV uptake by Gli36 cells. (**a**) Gli36-PalmGFP cells were treated with 293T-PalmtdTomato⁺ EVs and centrifuged at 4 °C for 1.5 h followed by a wash with PBS and replaced with EV-depleted media in the presence or absence of heparin (200 μg/mL). Cells were then collected at 0, 1 and 3 h to assess EV uptake by FACS analysis. (**b, c**) FACS analyses of Gli36-PalmGFP cells in the absence (**b**) or presence (**c**) of heparin.

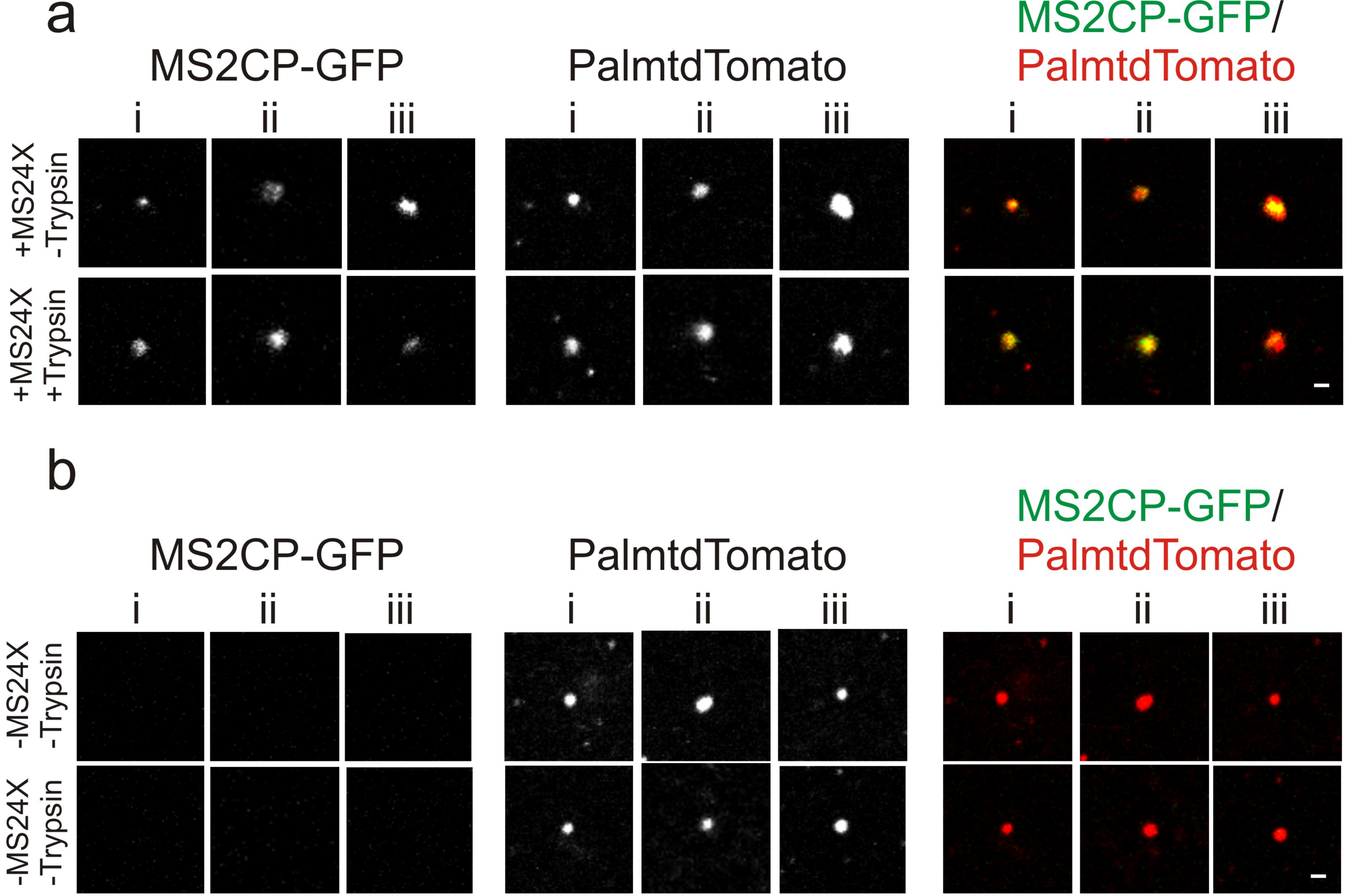

Supplementary Figure 5. Trypsin does not remove MS2CP-GFP from EVs. (**a, b**) EVs (subpanel i-iii) isolated from Gli36 cells co-expressing MS2CP-GFP and PalmtdTomato-MS24X (**a**) or PalmtdTomato (**b**) were imaged in the presence or absence of trypsin. Bar, 1 μm. MS2CP-GFP signals were detected with PalmtdTomato^{+} EV in the presence and absence of trypsin when the MS24X tag is present. No appreciable MS2CP-GFP signal was detected with PalmtdTomato^{+} when the MS24X tag is absent in the EV donor cells.

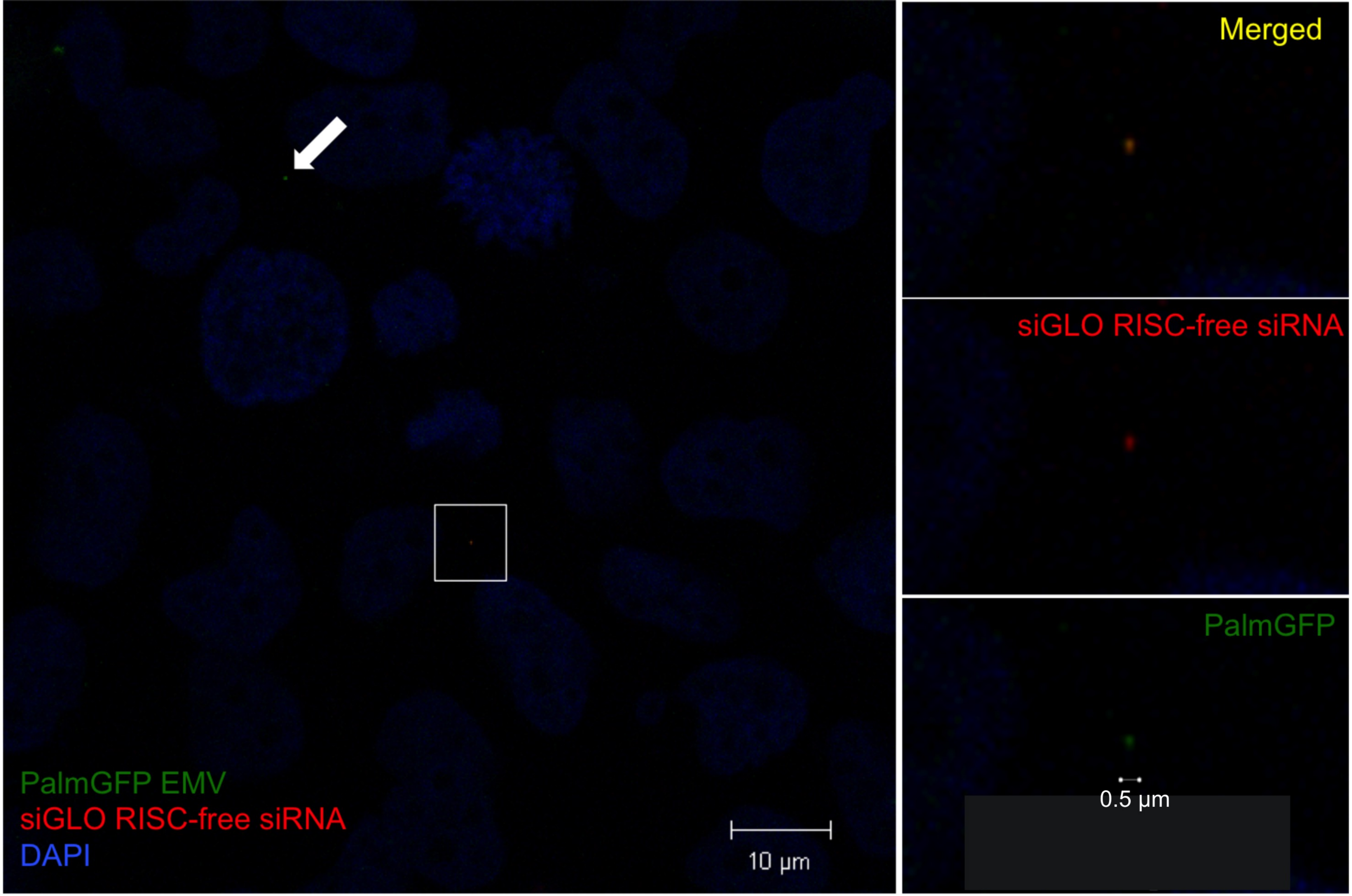

Supplementary Figure 6. Visualizing and tracking EV-delivered siRNAs. Gli36 glioma cells were seeded in the bottom dish of a transwell setup to serve as EV recipient cells. As EV donors, 293T-PalmGFP cells were transfected with siGLO RISC-free siRNA conjugated with DY-547, seeded into the upper transwell chamber (connected to the lower chamber through 1 μm pores) and allowed to incubate for 24 h. Gli36 cells were then fixed, stained with DAPI to reveal nuclei, and imaged with confocal microscopy. *Square box*, co-localization between PalmGFP$^+$ EVs and siGLO RISC-free siRNA in recipient cells. *Arrow*, imaging negative control for DY-547 illustrating only PalmGFP$^+$ EV signals. *Right panels*, magnified images showing PalmGFP$^+$ EV associated siRNAs.

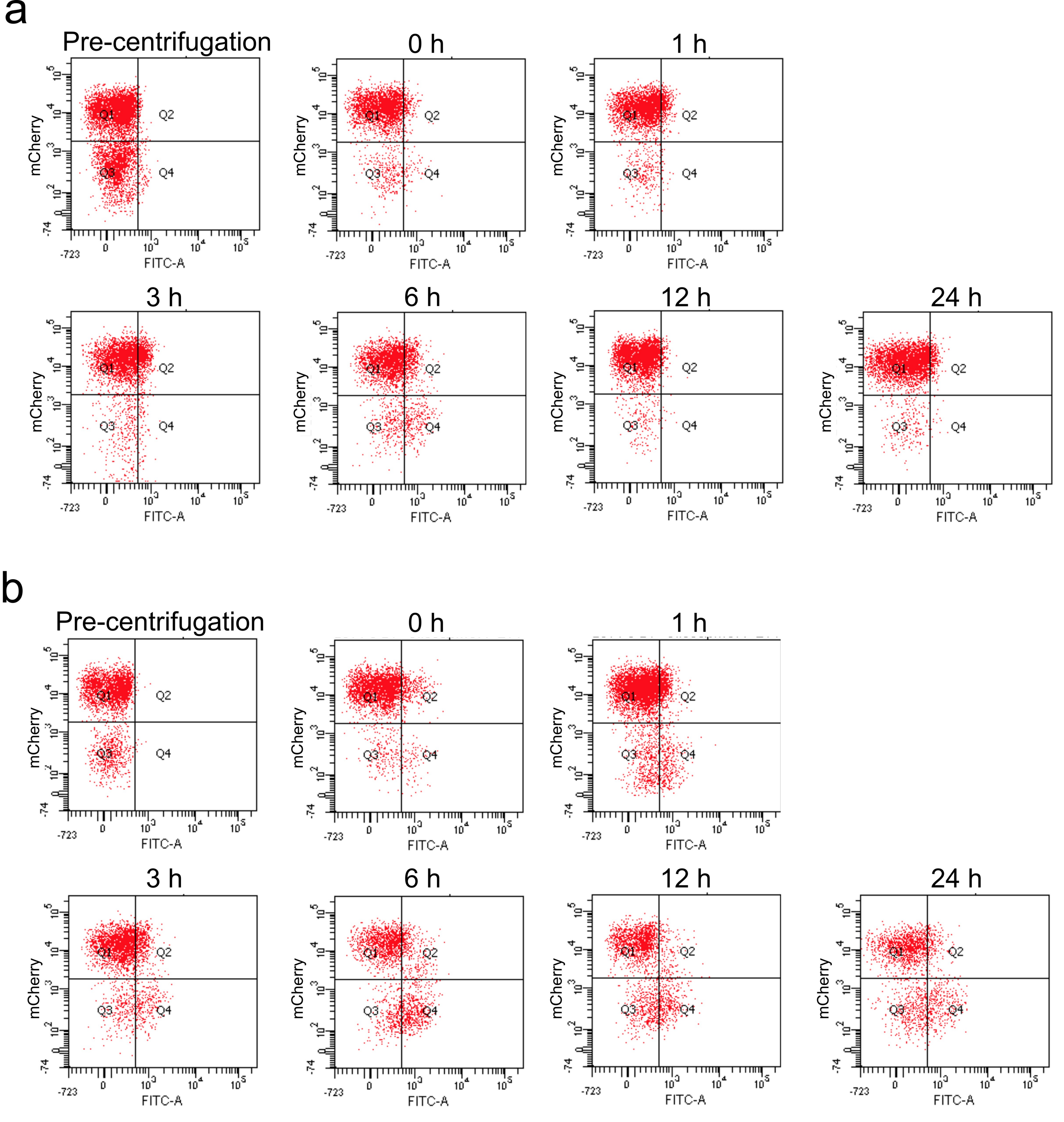

Supplementary Figure 7. FACS Analysis of PalmGFP^{+} EV uptake by Gli36-mCherry GBM cells. Gli36 cells stably transduced with mCherry were treated with PalmGFP+GlucB^{+} EVs (as described in legend to Fig. 6) in the presence (**a**) or absence (**b**) of CHX (20 µg/ml). Cell samples were analyzed by FACS at different time points from pre-centrifugation (4°C) to 24 h post-centrifugation (37°C).

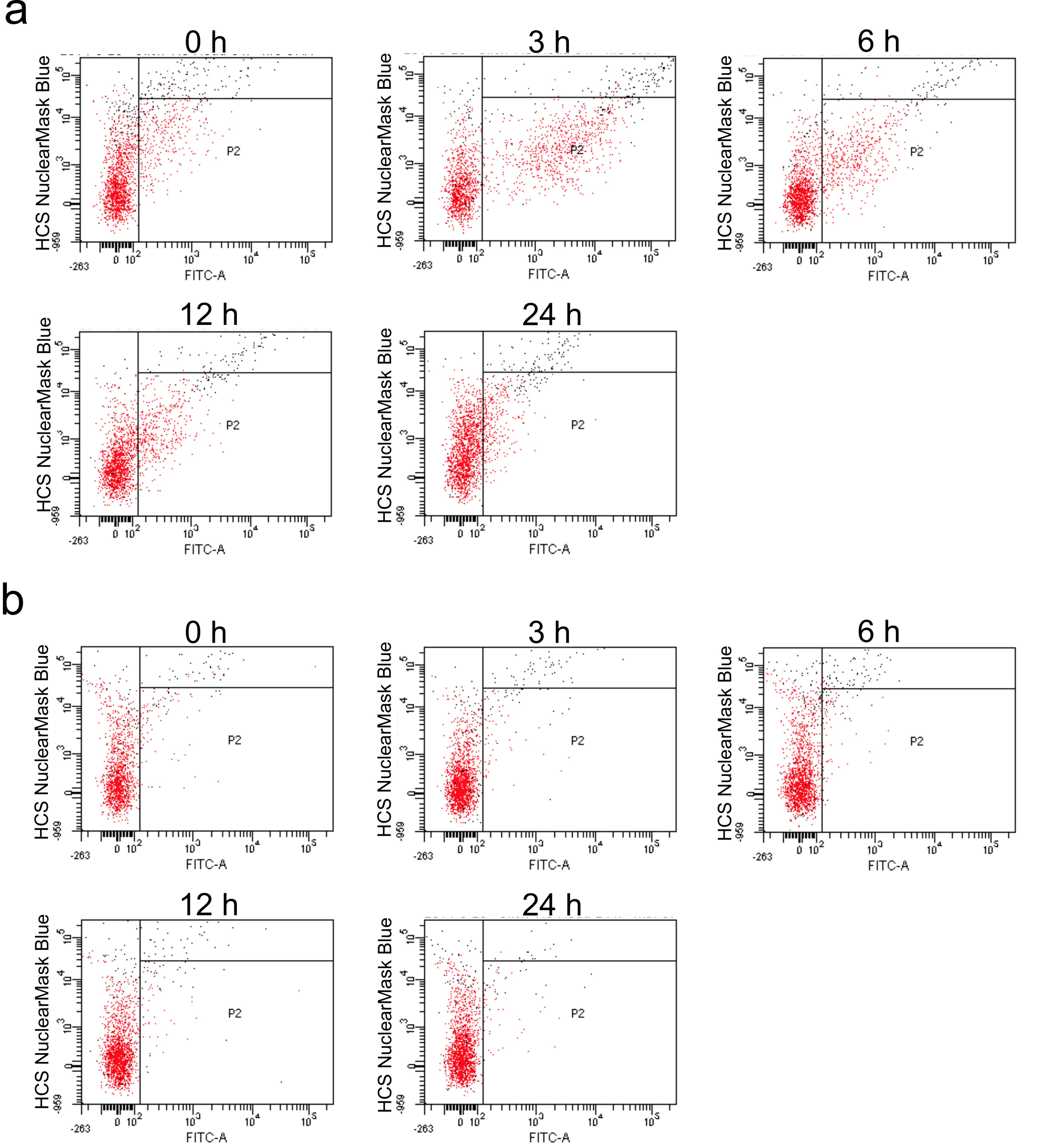

Supplementary Figure 8. FACS Analysis of nascent protein synthesis. Gli36 glioma cells stably transduced with mCherry exposed to PalmGFP+GlucB$^+$ EVs (as described in legend to Fig. 6) were treated with (**a**) or without (**b**) CHX (20 μg/ml) at 37°C. At each time point, cells were incubated with Click-iT® HPG for 30 min, collected for reaction with Alexa Fluor® 488 azide, and nascent protein synthesis was measured by FACS analysis.

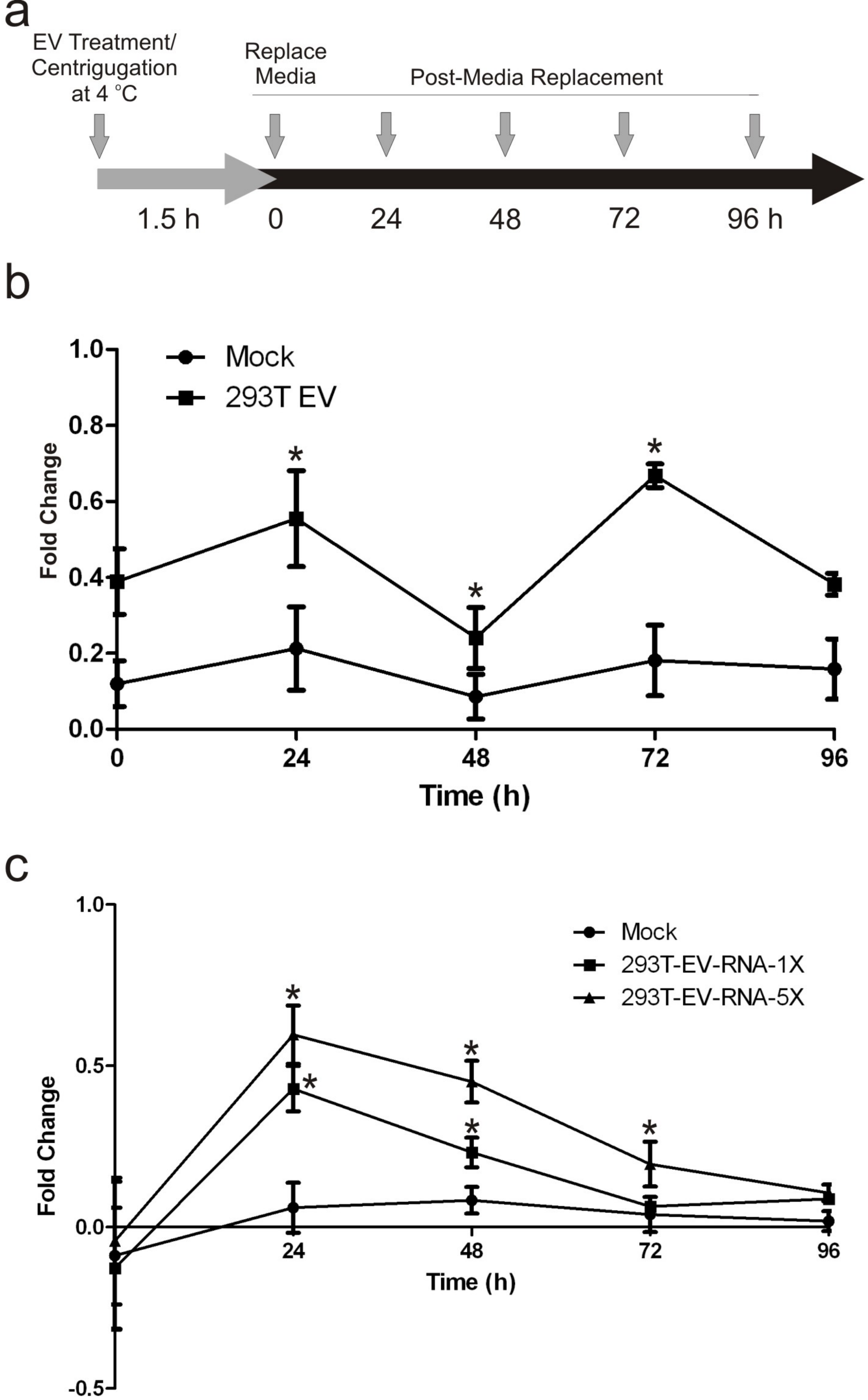

Supplementary Figure 9. EV and EV-RNA increase NFkB activity in EV recipient cells. (**a**) Schematic of EV treatment and sample collection time to assess NFkB-Gluc activity. PBS (control) or EVs isolated from wildtype 293T cells were added to 293T cells stably expressing NFkB-Gluc followed by centrifugation at 4 °C for 1.5 h. The EV-containing media was then removed, washed with PBS and replaced with EV-depleted media. Conditioned media was collected at indicated time points to assess NFkB activity (luciferase activity) of EV-treated cells. (**b**) NFkB-Gluc assay of EV-treated 293T cells showed a significant increase in NFkB activity starting at 48 h and up to 72 h post-EV treatment. *$p<0.05$ by paired t test. (**c**) Transfection of RNA isolated from 293T-EVs into 293T-NFkB-Gluc cells induced a robust increase in NFkB activity starting at 24 h and up to 72 h post-transfection. 1X represents the amount of RNA (54 ng) isolated from the volume of EVs used to treat 293T-NFkB-Gluc in (**b**). 5X indicates 5-fold more RNA (270 ng) than the 1X used for the transfection. Mock transfected samples were used as a control for Lipofectamine® 2000 transfection-induced NFkB activity while untransfected cells were used as baseline NFkB-Gluc activity for normalization. *$p<0.05$ by one way analysis of variance with Tukey's *post hoc* test when compared to the control (mock).

Inserts	Sense/Antisense
PalmGFP	5’ – ACCGGTCCACCATGCTGTGCTGTA – 3’ /<br>5’ - AATTCTCGAGGGGCCCTTACTTGTACAGCTCGTC – 3’
PalmtdTomato	5’–<br>ACCGGTCCACCATGCTGTGCTGTATGAGAAGAACCAAACAGG<br>TTGAAAAGAATGATGAGGACCAAAAGATCATGGTGAGCAAG<br>GGCGAG – 3’/<br>5’ – AATTCTCGAGGGGCCCTTACTTGTACAGCTCGTC – 3’

Supplementary Table 1. List of Primers
